# Targeting BRD4 prevents acute gouty arthritis by regulating pyroptosis

**DOI:** 10.7150/ijbs.46153

**Published:** 2020-10-17

**Authors:** Kun Hao, Wenjiao Jiang, Mengze Zhou, Hanwen Li, Yadong Chen, Fei Jiang, Qinghua Hu

**Affiliations:** 1 State Key Laboratory of Natural Medicines, Key Laboratory of Drug Metabolism and Pharmacokinetics, China Pharmaceutical University, Nanjing 210009, PR China; 2 School of Pharmacy, China Pharmaceutical University, Nanjing 211198, PR China; 3 School of Science, China Pharmaceutical University, Nanjing 211198, PR China

**Keywords:** BRD4, Acute gouty arthritis, Pyroptosis, NLRP3 inflammasome

## Abstract

**Background:** Acute gouty arthritis is a common inflammatory arthropathy resulting from urate deposition in joints during persistent hyperuricemia. Nevertheless, effective therapeutic strategies are still unavailable. Here, we propose the crucial role of bromodomain-containing protein 4 (BRD4) in acute gouty arthritis.

**Methods:** Therapeutic effect of BRD4 specific inhibitor JQ-1 on acute gouty arthritis was evaluated in vivo and in vitro. Pyroptosis was analyzed by Caspase-1/PI double staining and cleavage of gasdermin D (GSDMD). Expression of key factors involved in BRD4/NF-κB/NLRP3/GSDMD signaling pathway were measured by western blot, and colocalization of NLRP3 and ASC was detected using immunofluorescence. In addition, the role of BRD4 on monosodium uric acid crystals (MSU)-induced pyroptosis was verified in BRD4 siRNA-transfected THP-1 cells.

**Results:** Pretreatment of JQ1 and BRD4 siRNA significantly suppressed pyroptosis and inhibited activation of p65 NF-κB signaling as well as NLRP3 inflammasome in THP-1 cells exposed to MSU. In vivo, JQ-1 administration could effectively attenuate joint swelling and synovial inflammation in rats treated by intra-articular injection of MSU. More importantly, MSU led to macrophage pyroptosis and Brd4/NF-κB/NLRP3/GSDMD signaling induction in rat synoviums, which was improved by JQ-1.

**Conclusions:** Our study identifies the role of BRD4 in MSU-induced pyroptosis through regulating NF-κB/NLRP3/GSDMD signaling pathways, which provides a potential target for treatment of acute gouty arthritis.

## Introduction

Gout is regarded as a common inflammatory arthropathy characterized by hyperuricemia and accumulation of monosodium urate crystals (MSU) in joints 1. Deposition of MSU caused by the excessive serum uric acid levels could trigger an intense inflammatory process with pain along with tophi, leading to exaggeration of gouty inflammation 2. MSU crystallization-induced gout can occur in joints, peri-articular tissues and kidneys 3. Among them, joints are frequently subjected to gouty attacks, eventually resulting in acute gouty arthritis. Thus, articular injection of MSU is usually applied to establish the animal models to mimic acute gouty arthritis as previous studies shown 4.

At present, it has been reported that MSU administration leads to excessive activation of NLRP3 inflammasome in pathologies 5. Assembly of NLRP3 inflammasome depends on combination of Nod-Like Receptor Protein 3 (NLRP3), and apoptosis associated speck like protein (ASC), as well as cleavage of cysteinyl aspartate specific proteinase-1 precursor (pro-Caspase-1), which functions as pattern recognition receptors in response to stimuli 6. The secretion process of inflammation cytokines IL-1β is mediated by the NLRP3 inflammasome activation governing the following activation of Caspase-1 to promote the maturation of pro-IL-1β 7, 8. Notably, Caspase-1 activation is found to break the linker between the amino-terminal gasdermin-N and carboxy-terminal gasdermin-C domains in gasdermin D (GSDMD), leading to formation of membrane pores, which has been regared as a key step of pyroptosis 9-11. Pyroptosis processing could be observed, in which cell edema, secretion of pro-inflammatory intracellular events and pore formation in the cell membrane once pyroptosis started 12.

As a representative member of the BET (Bromodomain and Extra-Terminal) protein family, the bromodomain-containing protein 4 (BRD4) has been reported to mediate regulation of NF-κB signaling via acetylated RELA 13. Current studies indicated that small-molecule BRD4 inhibitor JQ-1 ameliorated rheumatoid arthritis via blocking NF-κB activation 14, 15. However, it is still unclear about the mechanism behind the protective effect of JQ-1 on regulating synovial inflammation. Here, we uncovered a role for bromodomain-containing protein 4 (BRD4) in acute gouty arthritis and explored how it is involved in the pathogenesis of MSU-induced acute gouty arthritis models.

## Methods and materials

### Reagents

JQ-1 and uric acid were purchased from Sigma-Aldrich (St. Louis, USA). Bicinchoninic acid Protein Quantification Kit was supplied by Vazyme Biotech (Nanjing, China). Interleukin-1β (IL-1β) enzyme-linked immunosorbent assay kit was provided by R&D Systems (Minneapolis, MN, USA). Primary antibodies were listed in Tables. Secondary antibodies conjugated with Alexa Fluor® 488, Alexa Fluor® 568, and Alexa Fluor® 647 were purchased from Abcam (Cambridge, UK). FLICA® 660 in vitro Caspase-1 Detection Kit was purchased from ImmunoChemistry Technologies (Bloomington, USA).

### Animals

30 Male Adult Sprague-Dawley rats (250-280g) were obtained from Shanghai SIPPR-BK Lab Animal Co. Ltd. and were allowed to acclimate to the laboratory conditions for one week prior to experiments. All animal studies were performed according to the Guide for the Care and Use of Laboratory Animals and approved by the China Pharmaceutical University Animal Ethics Committee.

### Preparation of MSU crystal

MSU crystal was prepared as our previous study 16. The uric acid solution was dissolved in double-distilled water at room temperature, followed by crystal formation at 4˚C overnight. After filtration, the precipitate was then dried at 70˚C for 4 h, grounded into a fine powder, and sterilized by heating at 180˚C for 2 h. The MSU crystals were suspended using sterile PBS for administration.

### Animal models and treatment regimes

To investigate the anti-inflammatory activity of JQ-1 in vivo, we establish a gouty arthritis model in rat by MSU injection, which has been widely used to apply in studies of acute gouty arthritis 16. Male adult Sprague-Dawley rats were randomly allocated to three groups: (1) Control group, (2) Model group, (3) JQ-1 group. The dose of JQ-1 was decided based on our preliminary experiments and the published studies 17. 1 h after pretreatment of JQ-1, animals were injected with 100 μl MSU crystals (500 μg/ml) dissolved in sterile saline in ankle joint. After MSU stimulation, the circumference of ankle joint was measured at 0, 2, 4, 8, 12, 24 h. Mechanical hyperalgesia assay was performed by Von Fray assay 18. After animals were sacrificed, synovium samples were collected for following analysis.

### Hematoxylin-eosin (H&E) staining

The synovium samples were fixed in the 4% paraformaldehyde solution immediately, and then embedded with paraffin. Subsequently, 5 μm paraffin sections were dewaxed, rehydrated, and stained with H&E according to the protocol as our previous study 19. After that, the histopathological evaluation was carried out.

### Cell Culture

THP-1 cell line (human myeloid leukemia mononuclear cells) purchased from American Type Culture Collection (ATCC, USA), were maintained at 37 °C under an atmosphere of 5 % CO_2_ in RPMI1640 medium containing 10% fetal bovine serum (Gibco), 100 IU/ml penicillin, and 100 IU/ml streptomycin. THP-1 cells were performed followed by pre-differentiation for 48 h in supplemented RPMI 1640 containing 100 ng/ml phorbol 12-myristate 13-acetate (PMA) (Sigma-Aldrich). Then, the matured THP-1 cells were incubated under JQ-1 (1 μM) for 1 h, followed by the stimulation of MSU (500 μg/ml) for 12 hours prior to the following analysis.

### Transfection

BRD4 siRNA (Genepharma, Shanghai) was used to transfect THP-1 cells at a confluency of 70%-90% using Lipofectamine 2000 (Life Technologies), according the manufacturer's instructions. After MSU stimulation for 12 h, cells were collected for the future analysis.

### IL-1β assay

The levels of IL-1β in rat synovial tissue homogenate and THP-1 cell supernatant were examined with enzyme-linked immunosorbent assay kit (R&D, USA) according to the manufacturer's instructions.

### Pyroptosis assay

Active Caspase-1 and PI fluorescence in vivo and in vitro are necessary for pyroptosis characterization. We measured the active caspase-1 level in cell suspensions with Caspase-1 Detection Kit (ImmunoChemistry Technologies, USA) using flow cytometry according to the manufacturer's instructions. And membrane pores induced by pyroptosis were marked by propidium iodide staining (BD, USA).

### Western blot

The synovium samples and THP-1 cells collected from each group were taken to western blotting experiment. Briefly, samples were lysed in a RIPA buffer and the supernatant was collected after centrifugation. The extracted protein was separated by sodium dodecyl sulphate-polyacrylamide gel electrophoresis (SDS-PAGE) and transferred to polyvinylidene difluoride (PVDF) membranes (Millipore, USA), followed by membrane blocking with 5% milk dissolved in Tris Buffered saline Tween (TBST) solution for 2h at room temperature. Primary antibodies incubation was performed at 4 °C overnight, as listed in Table 1. Membranes received a 2h incubation with horseradish peroxidase-conjugated secondary antibodies, and then bands detection was showed with Image Lab software (Bio-Rad, USA).

### Immunofluorescence

The collected synovial tissues were fixed, embedded and sectioned. After MSU exposure, the THP-1 cells were fixed with 4% paraformaldehyde solution for 20-30 min. Subsequently, permeabilization was carried out with Triton X-100 for 30 min, followed by incubation with blocking solution and primary antibodies at a 1: 50 dilution at 4 °C overnight. Tissue as well as cell samples were then incubated with secondary antibodies conjugated with Alexa Fluor dye at a 1:200 dilution, as listed in Table 2. After DAPI staining, fluorescent images were visualized usinga confocal laser scanning microscope (LSM 700, Zeiss, CA, USA).

### Statistical analysis

Data have been presented as mean values ± standard deviation. One-way analysis of variance (ANOVA) with Tukey multiple comparison test is used for data analysis, with p value < 0.05 considered to be significant.

## Results

### Pyroptosis processing shifts under BRD4 inhibition in vitro

**Figure 1:**
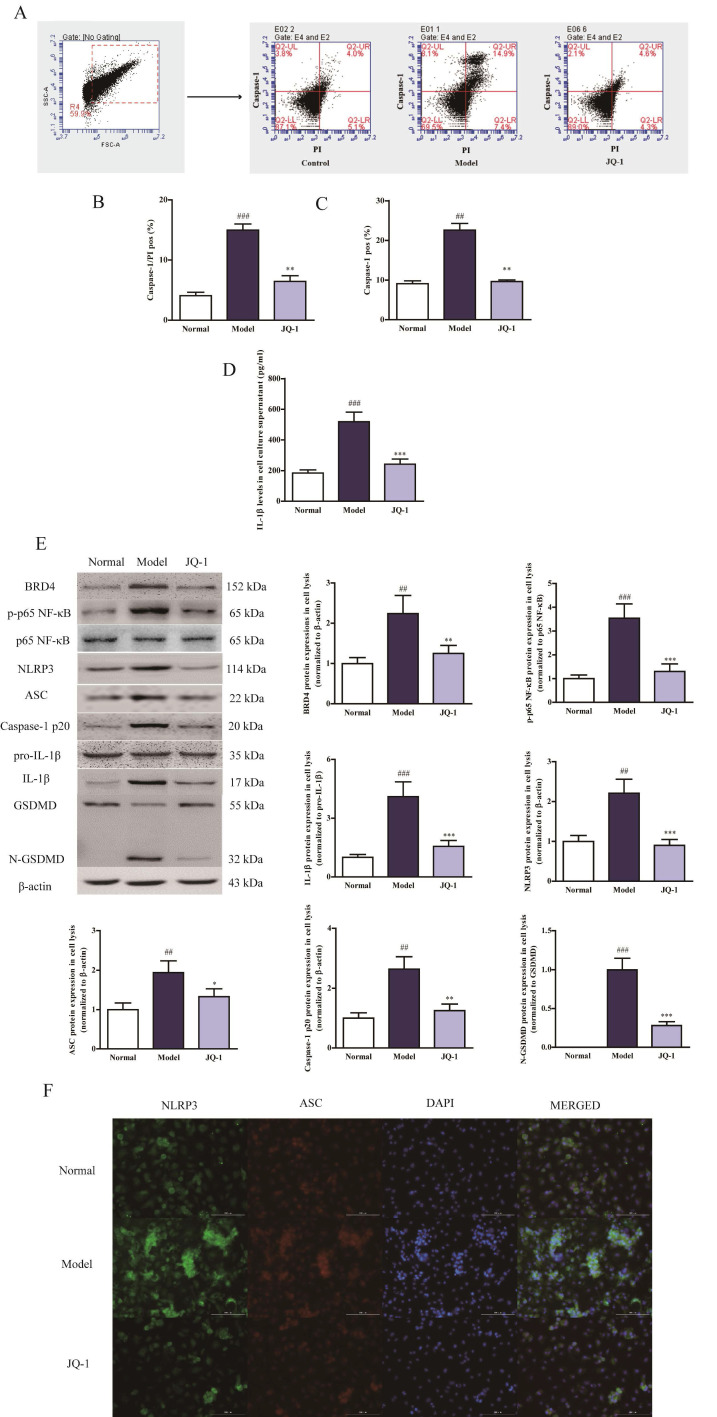
Pyroptosis processing shifts during BRD4 inhibition in MSU-induced THP-1 Human Promonocytes. After PMA treatment, the THP-1 cells were incubated with the absence or presence of serum free medium containing JQ-1 (1 μM) for 1 h, followed by the stimulation with MSU (500 μg/ml) for 12 h. The rate of pyroptotic cell death was examined with PI and active Caspase-1 double staining by flow cytometry (A). Positive Caspase-1/PI fluorescence intensity of samples in THP-1 cells (B). Positive Caspase-1 fluorescence intensity of samples in THP-1 cells (C). The culture supernatants level of IL-1β was detected using the ELISA kit (D). The expression of BRD4 and NLRP3 inflammasome activation was measured by western blotting (E). The relative optical densities of specific proteins were recorded. Representative confocal microscopy photographs of THP-1 cells with immunofluorescence changes are presented (F). NLRP3 protein was marked with the donkey anti-goat polyclonal secondary antibody conjugated with Alexa Fluor® 488 (Green). ASC protein was marked with the donkey anti-rabbit polyclonal secondary antibody conjugated with Alexa Fluor® 647 (Red). The data was presented as means ±SDs. Compared with normal group: ^#^P<0.05, ^##^P<0.01, ^###^P<0.001. Compared with Model group: ^*^P<0.05, ^**^P<0.01, ^***^P<0.001. Each group (n=4).

It is firstly reported that BRD4 inhibition could contribute to the acute gouty arthritis therapy with the regulation of pyroptosis processing. As a novel programmed cell death, pyroptosis has been recognized to be dependent on Caspase-1 activation and facilitate membrane disruption with positivity for propidium iodide (PI) staining 19. First of all, we conducted the pyroptosis assay in the MSU-induced THP-1 cells to assess if BRD4 inhibitor JQ-1 could suppress the onset of pyroptosis. Pyroptotic cells exhibited PI and Caspase-1 double positivity in flow cytometry detection 20. FLICA 660-YVAD-FMK was used to quantify active caspase-1 and PI dye was used to mark cells with membrane pores. MSU exposure induced a significant elevation in the amount of double positive cells in comparison with normal ones (Figure 1A-1C). And aforementioned alterations were reversed by the addition of JQ-1, meanwhile, the negative correlation with Caspase-1 activation could also be verified by the reduction of IL-1β secretion under BRD4 suppression, suggesting the effect of BRD4 inhibition works against MSU-induced pyroptotic cell death (Figure 1D).

### NLRP3 inflammasome activation occurs during MSU-induced pyroptosis

It has been demonstrated by emerging evidence that a positive correlation exists between the progression of gouty arthritis and NLRP3 inflammasome activation 21. Considering the up-stream role of NLRP3 inflammasome activation for caspase-1 eruption, corresponding analyses were performed to further investigate whether NLRP3 inflammasome was involved in the JQ-1 resistance to MSU-challenged pyroptosis in vitro 6. MSU caused the up-regulation of BRD4, phosphorylation of p65 NF-κB, induction of NLRP3 inflammasome followed by cleavage of GSDMD in THP-1 cells compared with normal group, which were obviously ameliorated by JQ-1 intervention, indicating that JQ-1 could influence the inflammatory response by mediating the upstream NLRP3 inflammasome inactivation (Figure 1E). As shown in Figure 1F, immunofluorescence data also confirmed JQ-1 blocked the assembly of NLRP3 inflammasome characterized by less co-localization of NLRP3 and ASC. Moreover, lower fluorescence intensity of NLRP3, ASC exhibited after JQ-1 pretreatment in MSU-induced THP-1 cells, implicating that blocking BRD4 expression could prevent the Caspase-1-dependent pyroptosis progression via inhibiting NLRP3 inflammasome activation.

### The effect of JQ-1 on acute gouty arthritis in vivo

**Figure 2:**
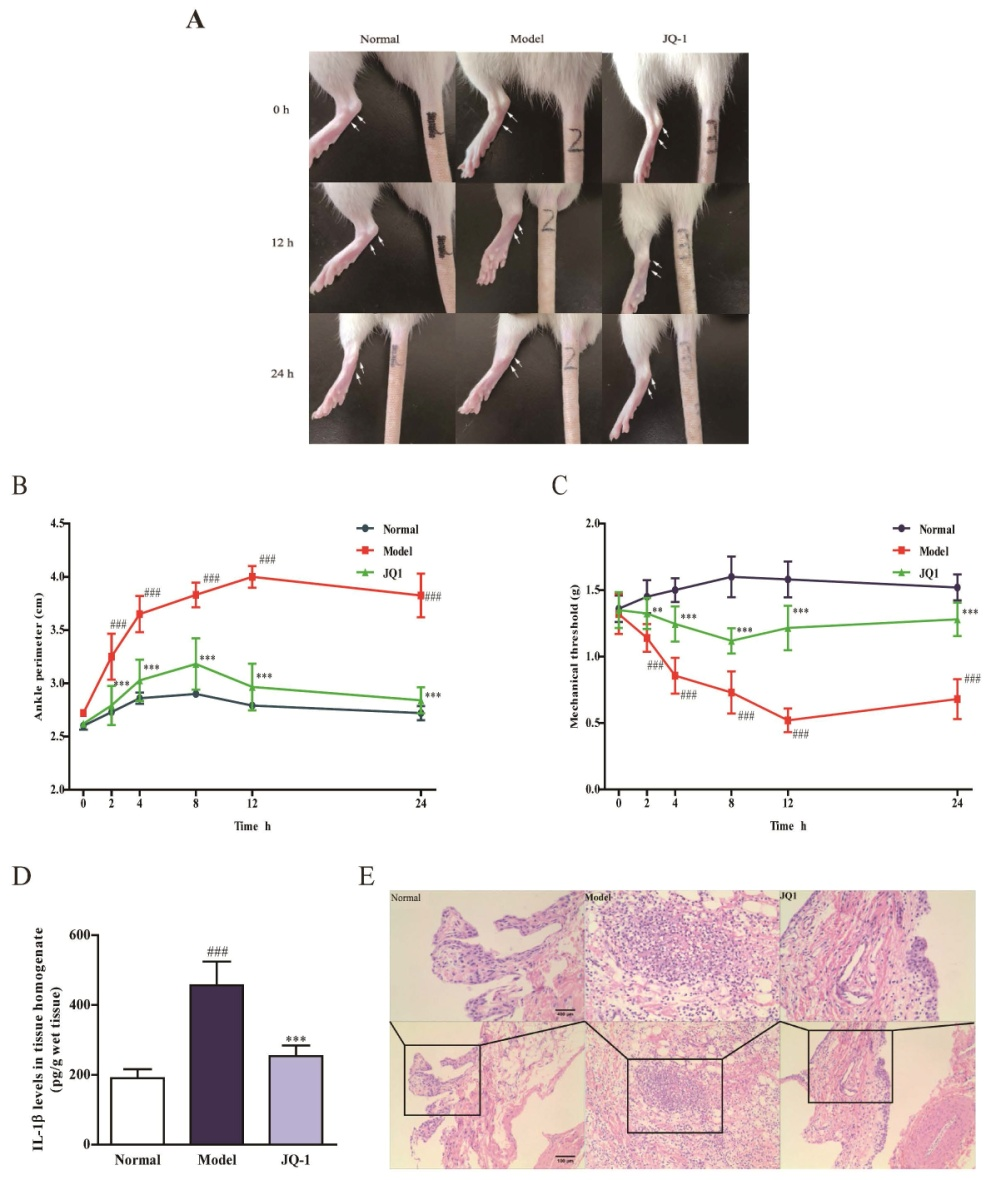
The effect of BRD4 inhibitor on MSU-induced acute gouty arthritis in vivo. 1 h after pretreatment of JQ-1, animals were injected with MSU crystals to induce acute gouty arthritis. Representative photographs to show the swelling of joints are presented (A). The injected ankle joint circumference of each rat was determined at 0, 2, 4, 8, 12, 24 h after MSU stimulation (B). Mechanical hyperalgesia, observed as an increase in nociceptive response, was assessed by Von Fray assay at 0, 2, 4, 8, 12, 24 h after MSU stimulation (C). The IL-1β level in synovium homogenate was detected by the ELISA kit (D). Representative photographs of histopathologic changes in synovium are presented (E). The data was presented as means ± SDs. Compared with Control group: ^#^P<0.05, ^##^P<0.01, ^###^P<0.001. Compared with Model group: ^*^P<0.05, ^**^P<0.01, ^***^P<0.001. Each group (n=6).

After pretreatment with JQ-1, joint circumference detection was performed at 0, 2, 4, 8, 12, 24 h after MSU crystals injection to evaluate the severity of synovial pathology. As revealed in Figure 2A-2C, joint circumference in model group significantly increased at 2, 4, 8 h and enlarged to peak value at 12 h point than those of normal group, indicating the enhanced joint swelling in response to MSU stimulation. On the contrary, BRD4 inhibitor restored the joint circumference to normal level with merely 1.05-fold than those of normal group. In comparison with normal ones, mechanical withdrawal of model rats declined rapidly at 2, 4, 8 h time points and decreased to 0.52 g at 12 h after MSU injection. This MSU-induced tendency of pain reaction was apparently reversed to some extent by administration of JQ-1 at different time points. Meanwhile, it was also confirmed by histopathological observation, evidenced by the suppressed synovial hyperplasia and severe neutrophil infiltration of JQ-1 samples (Figure 2E). Additionally, an improvement of MSU-induced elevation of IL-1β concentration was found in JQ-1 rats, affirming the protection of JQ-1 from gouty arthritis (Figure 2D).

### BRD4 inhibitor prevents the pyroptosis death accompanied with suppressed NLRP3 inflammasome activation in vivo

**Figure 3:**
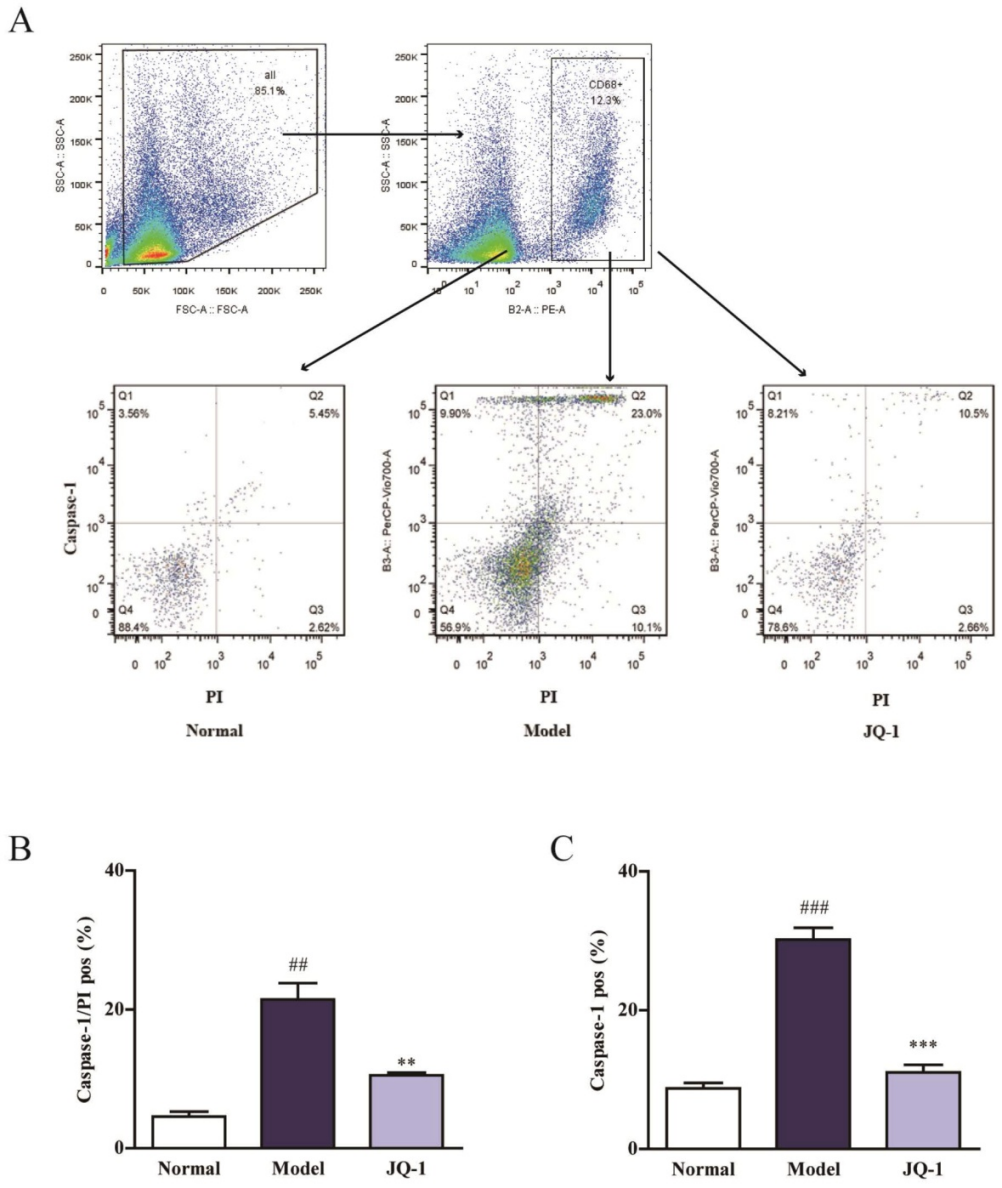
BRD4 inhibitor regulates the pyroptosis death in synovial tissue. 1 h after pretreatment of JQ-1, animals were injected with MSU crystals to induce acute gouty arthritis. To determine the rate of pyroptotic cell death in synovial tissue, macrophages were classified based on positive staining for CD68. Then pyroptosis assay was performed in the macrophages obtained from synovium using PI and active Caspase-1 double staining by flow cytometry (A). Positive Caspase-1/PI fluorescence intensity of samples (B). Positive Caspase-1 fluorescence intensity of samples (C). The data was presented as means ± SDs. Compared with Control group:^ #^P<0.05, ^##^P<0.01, ^###^P<0.001. Compared with Model group:^ *^P<0.05, ^**^P<0.01, ^***^P<0.001. Each group (n=4).

Considering the connection between IL-1β and Caspase-1, flow cytometry was used to investigate whether BRD4 inhibitor have an impact on Caspase-1-dependent pyroptosis in animal model of gouty arthritis (Figure 3A). Macrophages were classified based on positive staining for CD68. Interestingly, the quantity pyroptotic macrophages (double-positive) was remarkably elevated in synovial tissue of rats injected by MSU, which was pulled down closer to normal level by the intervention of JQ-1 (Figure 3B, 3C). And this inhibition of JQ-1 injection in the Caspse-1 activation in synovium lysates of model animals was confirmed using western blotting and immunofluorescence. Hence, we proposed that Brd4 expression might be involved in relative mechanism of pyroptosis.

**Figure 4:**
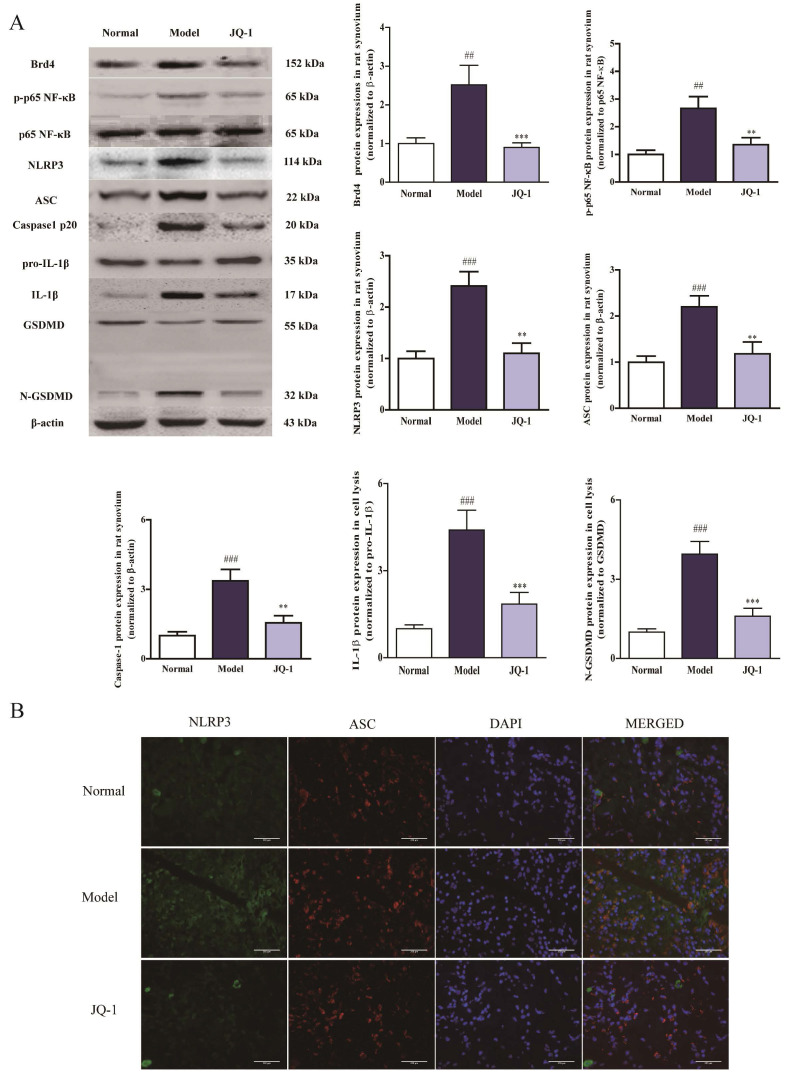
Suppressed NLRP3 inflammasome activation in acute gouty arthritis during Brd4 inhibition in acute gouty arthritis. 1 h after pretreatment of JQ-1, animals were injected with MSU crystals to induce acute gouty arthritis. The protein expression of Brd4 and NLRP3 inflammasome pathway in synovium after the JQ-1 treatment (A). The relative optical densities of specific proteins were recorded. NLPR3 inflammasome activation in synovial tissues was evaluated with immunofluorescence staining (B). NLRP3 protein was marked with the donkey anti-goat polyclonal secondary antibody conjugated with Alexa Fluor® 488 (Green). ASC protein was marked with the donkey anti-rabbit polyclonal secondary antibody conjugated with Alexa Fluor® 647 (Red). The data was presented as means ± SDs. Compared with Control group:^ #^P<0.05, ^##^P<0.01, ^###^P<0.001. Compared with Model group: ^*^P<0.05, ^**^P<0.01, ^***^P<0.001. Each group (n=4).

Pyroptosis has not been characterized in acute gouty arthritis pathologies, nor has its relevance to MSU-challenged in vivo model been elucidated. And it has been reported that NLRP3 inflammasome could induce this inflammatory cascade-related pyroptosis 22. Consistent with cell experiments, immunoblotting analysis revealed an apparent up-regulation in Brd4 expression, p65 NF-κB phosphorylation, NLRP3 inflammasome activation and cleavage of GSDMD under MSU exposure in synovium specimens. Conversely, the blockage of Brd4 by JQ-1 down-regulated these expressions in synovium, suggesting that the effect of JQ-1 on pyroptosis might be mediated by NLRP3 inflammasome signaling (Figure 4A). Besides, Figure 4B showed the JQ-1 treatment could block activated NLRP3 signaling after MSU stimulation, as evidenced by reduced fluorescence intensity of NLRP3, ASC and their co-localization in synovium (Figure 4B). The data above indicated that the formation of NLRP3 inflammasome complex was blocked in the presence of BRD4 inhibitor JQ-1, suggesting the role of BRD4 in pyroptosis was related to the NLRP3 signaling cascade.

### BRD4 knockdown is resistant to MSU stimulation in vitro

**Figure 5:**
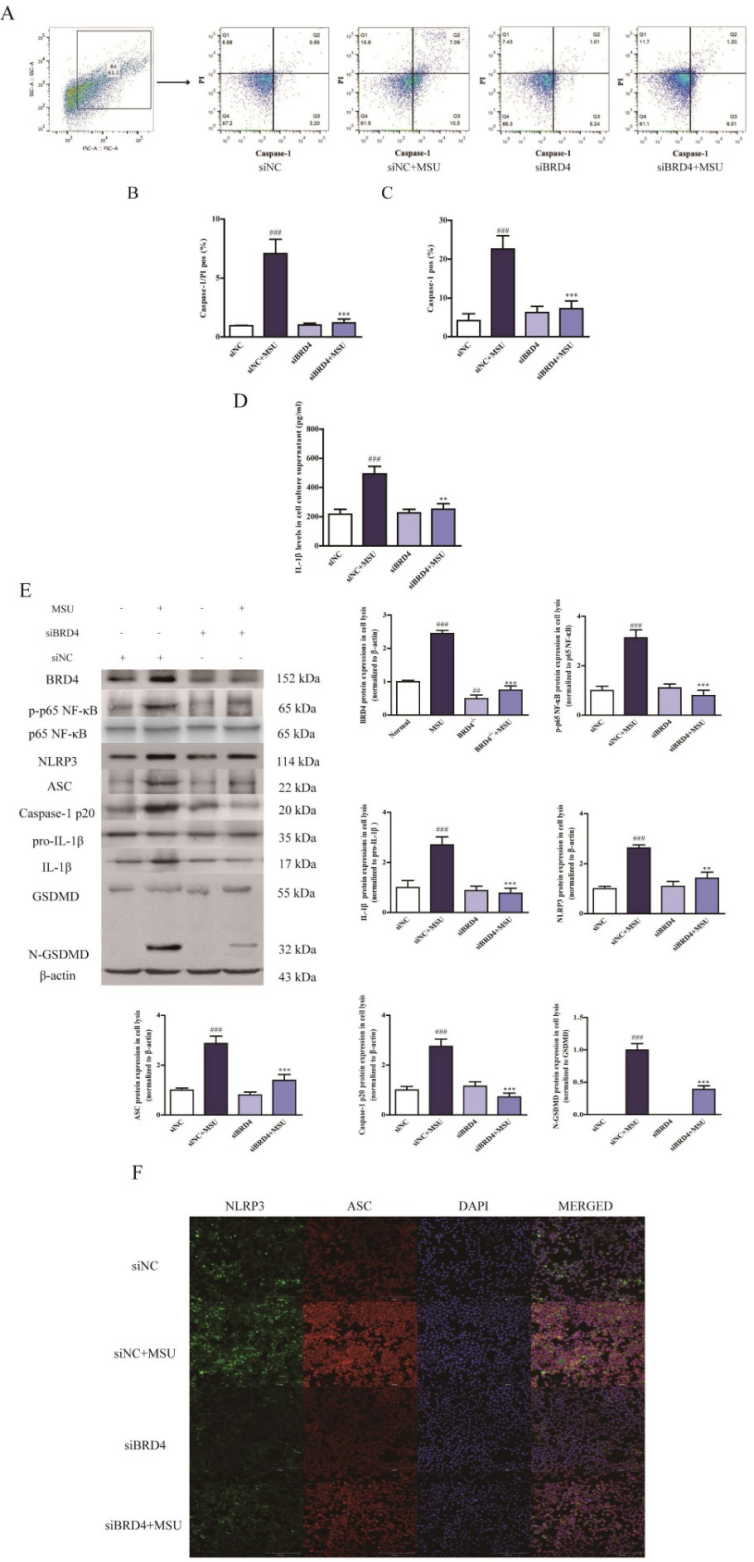
The effect of BRD4 knockdown on the pyroptosis assay. BRD4 siRNA was used to transfect THP-1 cells at a confluency of 70%-90% with Lipofectamine 2000. After transfection for 24 h, the THP-1 cells were stimulated by MSU (500 μg/ml) for 12 h. The rate of pyroptotic cell death was examined with PI and active Caspase-1 double staining by flow cytometry (A). Positive Caspase-1/PI fluorescence intensity of samples in THP-1 cells (B). Positive Caspase-1 fluorescence intensity of samples in THP-1 cells (C). The culture supernatants level of IL-1β was detected using the ELISA kit (D). The expression of BRD4 and NLRP3 inflammasome activation was measured by western blotting (E). The relative optical densities of specific proteins were recorded. Representative confocal microscopy photographs of THP-1 cells with immunofluorescence changes are presented (F). NLRP3 protein was marked with the donkey anti-goat polyclonal secondary antibody conjugated with Alexa Fluor® 488 (Green). ASC protein was marked with the donkey anti-rabbit polyclonal secondary antibody conjugated with Alexa Fluor® 647 (Red). The data was presented as means ± SDs. Compared with Control group: ^#^P<0.05, ^##^P<0.01, ^###^P<0.001. Compared with Model group: ^*^P<0.05, ^**^P<0.01, ^***^P<0.001. Each group (n=4).

To address the role of BRD4 in acute gouty arthritis, we examined pyroptotic response in BRD4 knockdown (KD) THP-1 cells. The stability of Caspase-1 activation and membrane integrity was observed in BRD4 knockdown THP-1 cells once MSU stimulated (Figure 5A-5C). In contrast, it was without BRD4 transfection that MSU stimulation could still augment pyroptosis reaction with the more potent positivity of Caspase-1 and PI staining, suggesting that the role of BRD4 in governing the regulation of pyroptosis onset. By the way, as shown in the Figure 5D, the secretion of IL-1β in cell supernatant was also unchanged in the presence of BRD4 KD cells under MSU exposure, which was consist with our hypothesis.

And we found the western blotting and immunofluorescence data uncovered the similar phenomenon in which BRD4 knockdown abolished relevant alterations of the p65 NF-κB phosphorylation, NLRP3 inflammasome activation and cleavage of GSDMD, which were brought by MSU administration, demonstrating that BRD4 could regulate the p65 NF-κB phosphorylation and its downstream signaling cascade- NLRP3 inflammasome function (Figure 5E, 5F). Notably, NLRP3 could directly recognize MSU as a danger signal and subsequently induce inflammatory response, which might contribute to the slight activation of NLRP3 in siBRD4+MSU group compare to siBRD4 alone.

## Discussion

Our study is the first research to reveal the role of BRD4 in MSU-induced NLRP3 inflammasome activation in acute gouty arthritis. Above results section demonstrated that enhanced NLRP3 activation caused by MSU crystals would lead to ankle joints swelling, severe synovial inflammation with an apparent neutrophilic infiltration and intense pyroptotic death progression characterized by the increased positivity of PI and Caspase-1 double-staining. Meanwhile, these effects were predominantly reversed through the pretreatment with BRD4 inhibitor JQ-1, suggesting its involvement of Caspase-1-dependent pyroptotic cell death in mechanisms of acute gouty arthritis.

MSU-induced pyroptosis, a kind of programmed cell death, is usually marked by activated caspase-1 and membranolysis, thus, double staining detection of caspase-1+PI has been used to evaluate of NLRP3-related pyroptosis, which has been regarded as dependable method 20. As a Capase-1-mediated programmed death, pyroptosis process is usually accompanied by pore formation of plasma membrane, followed by permeability alterations and subsequent cellular leakages. And PI can go through damaged plasma membrane and combine with DNA, but not complete membrane structure. Therefore, based on the selective caspase-1 detection, PI staining also contributed to evaluate the pyroptotic death. On the other hand, active Caspase-1 performed the cleavage of the amino-terminal gasdermin-N from GSDMD, which was necessary for pyroptosis 11. Therefore, we detected protein expression of GSDMD-N in order to determine whether cell would undergo pyroptosis. Once sensing according stimuli, after priming step, the relevant NLR (NOD-like receptors, NLRs) can oligomerize to assemble an inflammasome complex followed by the maturation of pro-Caspase-1 6. Subsequently, active Caspase-1 serves to cleave the pro-inflammatory cytokines IL-1β into its bioactive forms. Meanwhile, active IL-1β release could also promote a potent inflammatory response in pyroptosis 23. Currently, enhanced NLRP3 inflammasome could mediate caspase-1 activation, interleukin-1β secretion, and pyroptosis by the addition of NLRP3 stimuli, which contributed to the pathology of rheumatoid arthritis in vivo 24. And hepatocyte pyroptotic cell death has been reported as a novel mechanism of NLRP3 inflammasome-mediated liver damage 20. Our data suggest that NLRP3 inflammasome activation would also exaggerate the development of acute gouty arthritis, accompanied with pyroptosis eruption.

In general, NLRP3 protein must be primed in prior to NLRP3 inflammasome activation 6. Priming is required for NLRP3 inflammasome to increase the cellular NLRP3 level in response to stimulus, like, LPS binding to TLR4 25. Previous studies have reported the critical role of NF-κB in synovial inflammation 26, 27. NF-κB has been well known to serve as a mediator of inflammation and energy failure, which governs the production of pro-inflammatory cytokine 28. As the inhibitor of NF-κB, IκBα is dominated by IκB kinase (IKK) complex consisting of IKK-α and IKK-β. Upon stimulation, the NF-κB subunit p65 would be activated by the phosphorylation and degradation of the IκBα 29. In general, NF-κB is distributed in the cytosol binding to its inhibitor IκB. NF-κB separates from IκBα under inflammation, followed by the translocation into nucleus to regulate the transcription of pro-inflammatory cytokines, which will promote the activation of NLRP3 inflammasome signaling. In the current study, results showed the effect of BRD4 knockdown on the phosphorylation of NF-κB with unchanged IL-1β release in vivo and in vitro, confirming the relevance of BRD4 expression to NF-κB activation. Additionally, downstream NLRP3 inflammasome activation turned out to be invalid once BRD4 was pulled down, implying that NF-κB phosphorylation followed by NLRP3 inflammasome activation are crucial factor accounting for the protective effect of BRD4 inhibition under MSU stimulation.

**Figure 6:**
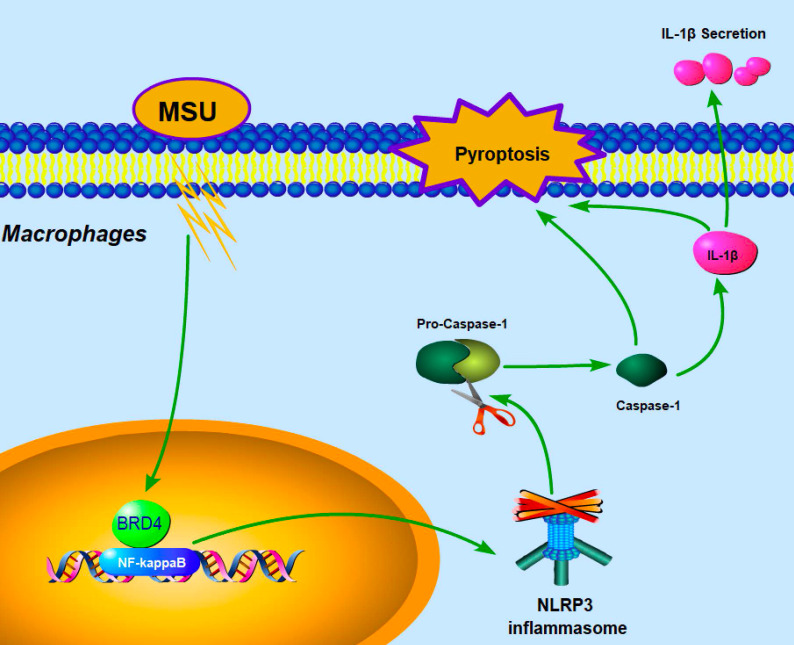
Proposed mechanism: BRD4 regulates NLRP3 inflammasome activation and IL-1β secretion during MSU stimulation.

Our current data demonstrated that BRD4 suppression could partially counteract the severity of pyroptotic death in acute gouty arthritis via the BRD4/NF-κB/NLRP3/GSDMD axis. Our proposed hypothesis about mechanisms was exhibited as shown in the Fig 6. Similarly, recent studies indicated the marked potency of BRD4 inhibitor JQ-1 against synovial inflammation by blocking IκB kinase-mediated NF-κB translocation in rheumatoid fibroblast-like synoviocytes 14, 15. Notably, affecting the acetylated histones recognition of the BET family proteins would attribute to regulate pro-inflammatory gene expression 30. And JQ1 was also found to prevent the “cytokine storm” in endotoxemic mice to rescue animals from LPS-induced inflammation, suggesting that targeting BET proteins with the small-molecule inhibitor could improve inflammatory conditions 31.

In conclusion, articular Brd4 expression was correlated with the joint lesion and pyroptosis cascade events in MSU-induced acute gouty arthritis. The protective effect of BRD4 inhibition by JQ-1 or siRNA on MSU-induced model might be mediated via the BRD4/NF-κB/NLRP3/GSDMD axis.

## Supplementary Material

Supplementary figure.

## Figures and Tables

**Table 1:** Antibodies used for Western blot analysis

Company	Description	Catalog number
BIOSS Biotech (Beijing, P. R. China)	Rabbit NLRP3 antibody	bs-6655R
	Rabbit ASC antibody	bs-6741R
	Rabbit caspase-1 antibody	bs-6368R
	Rabbit BRD4 antibody	bs-9759R
	Rabbit IL-1β antibody	bs-6319R
	Rabbit GSDMD antibody	bs-14287R
Cell Signaling Technology	Rabbit p65 NF-κB antibody	#8242
(Boston, MA, USA)	Rabbit p-p65 NF-κB antibody	#3033
Bioword Technology, Inc (Louis Park, MN. USA)	Rabbit β-actin antibody	AP0060
	Goat anti-rabbit IgG HRP	BS13278

**Table 2:** Antibodies used forimmunofluorescence

Company	Description	Catalog number
Santa Cruz Biotechnology, Inc (Dallas, TX, USA)	Goat NLRP3 antibody	sc-34410
	RabbitASC antibody	sc-22514-R
AbcamInc (Cambridge, UK)	Donkey Anti-Goat IgG H&L (Ex:495nm, Em:519nm)	Ab150129
	Donkey Anti-Rabbit IgG H&L (Ex:652nm, Em:668nm)	Ab150075
	Goat Anti-Mouse IgG H&L (Ex:578nm, Em:603nm)	Ab175473
