# Targeting BRD4 prevents acute gouty arthritis by regulating pyroptosis: Erratum

**DOI:** 10.7150/ijbs.107312

**Published:** 2025-01-01

**Authors:** Kun Hao, Wenjiao Jiang, Mengze Zhou, Hanwen Li, Yadong Chen, Fei Jiang, Qinghua Hu

**Affiliations:** 1 State Key Laboratory of Natural Medicines, Key Laboratory of Drug Metabolism and Pharmacokinetics, China Pharmaceutical University, Nanjing 210009, PR China; 2 School of Pharmacy, China Pharmaceutical University, Nanjing 211198, PR China; 3 School of Science, China Pharmaceutical University, Nanjing 211198, PR China

In our previous paper, the author noticed an error in Figure 2. As shown in Figure 2A in the article, due to our negligence in typesetting, we accidentally placed the representative images originally belonging to the 0 h of the Control group into the 12 h of the control group.

We rechecked the original data and made sure that this correction did not impact the results of our study as reported in the published paper, and maintained full confidence in the quality of the experiments and the conclusions drawn in the paper. All authors agree to the erratum.

We deeply regret the oversight that led to this error before manuscript submission, which was unfortunately not detected during subsequent review and publication stages. We sincerely apologize for any inconvenience this may have caused to our readers.

Figure 2 should be corrected as follows.

## Figures and Tables

**Figure 2:**
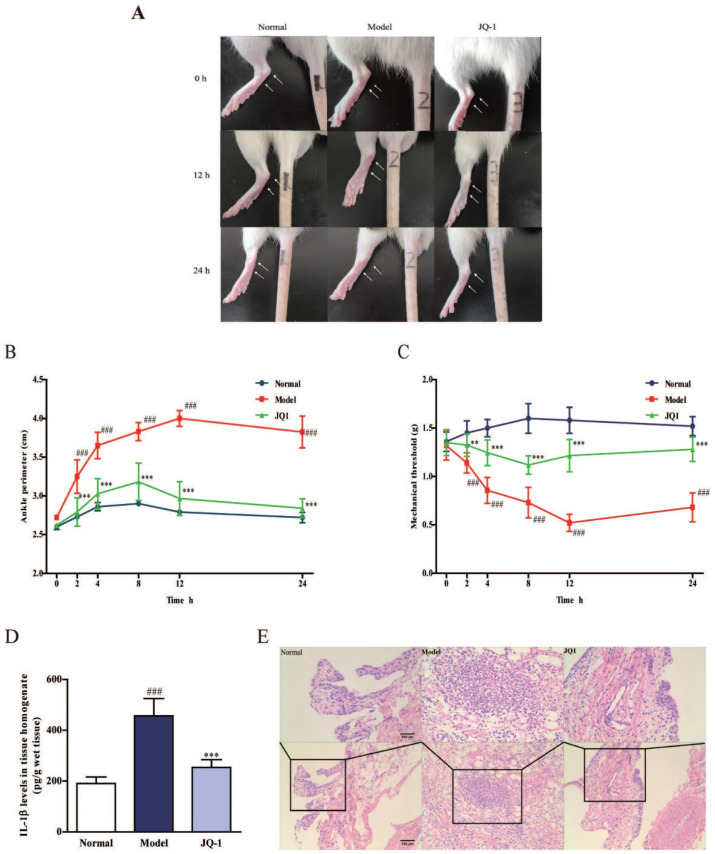
** The effect of BRD4 inhibitor on MSU-induced acute gouty arthritis in vivo.** 1 h after pretreatment of JQ-1, animals were injected with MSU crystals to induce acute gouty arthritis. Representative photographs to show the swelling of joints are presented (A). The injected ankle joint circumference of each rat was determined at 0, 2, 4, 8, 12, 24 h after MSU stimulation (B). Mechanical hyperalgesia, observed as an increase in nociceptive response, was assessed by Von Fray assay at 0, 2, 4, 8, 12, 24 h after MSU stimulation (C). The IL-1β level in synovium homogenate was detected by the ELISA kit (D). Representative photographs of histopathologic changes in synovium are presented (E). The data was presented as means ± SDs. Compared with Control group: ^#^*P*<0.05, ^##^*P*<0.01, ^###^*P*<0.001. Compared with Model group: ^*^P<0.05, ^**^P<0.01, ^***^P<0.001. Each group (n = 6).

