# Supplementary material for: Targeting BRD4 prevents acute gouty arthritis by regulating pyroptosis
Source: Int J Biol Sci. 2020 Oct 17;16(16):3163–73. doi: 10.7150/ijbs.46153 (PMC7645998; doi:10.7150/ijbs.46153)
Supplement: Supplementary file 1 — Supplementary figure. [file ijbsv16p3163s1.pdf]

Supplemental Information

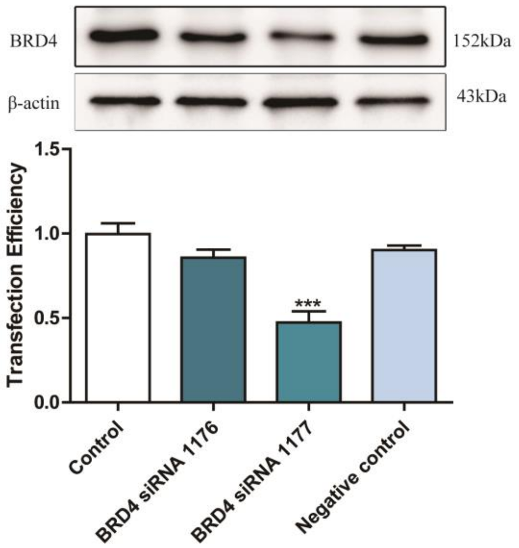

Fig. S1 BRD4 siRNA 1176 and BDR4 siRNA 1177 were added to THP-1 cells for 72 h. Then, cell lysates were used to perform western blot for BRD4 expression in THP-1 cells. Quantification of the protein level of BRD4 was expressed as the ratio (in percentage) of control group. The untreated THP-1 cells were used as a control. Data are shown as means ± SD (n=3). $^{***}P<0.001$ vs. control group.
